# *Notes from the Field*: Unexplained Dermatologic, Respiratory, and Ophthalmic Symptoms Among Health Care Personnel at a Hospital — West Virginia, November 2017–January 2018

**DOI:** 10.15585/mmwr.mm6844a2

**Published:** 2019-11-08

**Authors:** Todd J. Lucas, Mark Holodniy, Marie A. de Perio, Kiran M. Perkins, Isaac Benowitz, David Jackson, Ian Kracalik, Michael Grant, Gina Oda, Krista M. Powell

**Affiliations:** ^1^Epidemic Intelligence Service, CDC; ^2^Division of Healthcare Quality Promotion, National Center for Emerging and Zoonotic Infectious Diseases, CDC; ^3^National Institute for Occupational Safety and Health, CDC; ^4^Public Health Surveillance and Research, Office of Quality, Safety, and Value, Department of Veterans Affairs, Washington, DC.

During November 8–December 25, 2017, health care personnel at an 80-bed acute care facility in West Virginia reported dermatologic, respiratory, and ophthalmic symptoms to management or the occupational health clinic, prompting concern about a common exposure, possibly related to construction activities. Symptoms of affected staff members, who performed a range of clinical and nonclinical duties, often improved hours to days after leaving the hospital, suggesting potential exposure to an environmental irritant. Initially, hospital leadership encouraged symptomatic persons to seek evaluation at the occupational health clinic, although systematic evaluations were not implemented. No etiology was identified by environmental sampling for fibers, volatile organic compounds, or mold. In the absence of a clear etiology, hospital leadership stopped inpatient admissions, transferred inpatients from the two wards where most symptomatic staff members worked, and completed cleaning to include associated air-handling systems. Dermatology and allergy consultants evaluated symptomatic staff members, but because of varying clinical manifestations, results were inconclusive. On December 26, one of the closed wards reopened; during the ensuing week, six additional workers reported symptoms, and onsite CDC assistance was requested to identify an etiology. A CDC team arrived on January 8, 2018, and met with hospital and union leadership, reviewed occupational health records, observed occupational health encounters, performed unstructured individual interviews with both affected and unaffected health care personnel, assessed the physical environment, and reviewed environmental testing results. Despite these efforts, investigators were unable to identify an etiology, and the outbreak resolved without intervention.

CDC investigators found that during November 1, 2017–January 12, 2018, a total of 114 workers at the West Virginia hospital had 154 occupational health encounters, including 28 (25%) workers who had multiple encounters (Figure). The most frequently reported symptoms were rash (86%), upper respiratory or ophthalmic symptoms (e.g., nasal congestion and itchy eyes) (43%), and lower respiratory symptoms (e.g., cough and wheezing) (24%). Temperature, documented in 148 (96%) records, never exceeded 100.2°F (37.9°C). Records did not uniformly include symptom severity or duration, exposures, physical findings, or absenteeism. Interviews with a convenience sample of eight persons who had visited the hospital’s occupational health clinic with complaints described wide-ranging symptomatology often characterized as mild, and for which they otherwise would not have sought evaluation outside the investigation.

**FIGURE:**
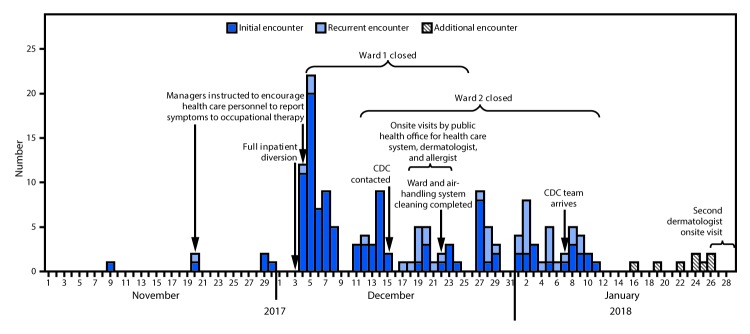
Number of occupational health encounters (N = 154) for dermatologic, respiratory, or ophthalmic symptoms among 114 hospital workers — West Virginia, November 1, 2017–January 26, 2018* * Systematic collection of data was not available during January 13–26, 2018. The “additional encounters” shown were not included in the data analyses.

Health care personnel reported that vigorous investigation and response and related effects (e.g., physical barriers and empty wards) heightened their concerns about workplace exposures. Multiple persons reported hearing rumors that occupational health evaluation would be required for subsequent compensation eligibility for potential occupational toxic exposure. Investigators identified no etiology. All units reopened January 16, 2018, and another dermatology consultant visit, including skin biopsy of a symptomatic staff member, occurred during January 26–29, 2018. The biopsy was nonspecific, and no other personnel reported symptoms after January 26, 2018. Despite no identified etiology or recognized interventions, the outbreak resolved.

Arrival of the CDC team 1 month after the peak in health encounters might have limited the ability to identify an etiology. However, inconsistent symptomatology, reports of persons seeking evaluation for subjectively mild symptoms, and rumors that future compensation might require seeking care suggest that response efforts might have inadvertently contributed to reports of illness. Outbreaks of unknown etiology perpetuated by response efforts have been described previously (*1*,*2*).

This investigation demonstrates challenges inherent in investigating outbreaks of unknown etiology and supports the hypothesis that response actions can heighten concern, potentially increasing reporting. Robust investigations might reinforce suspicions of concealed findings even in the absence of true pathology (*3*). Although clear communication and directed interventions are vital, such efforts could unintentionally potentiate events. Finally, this investigation highlights the need for a standardized clinical assessment tool, reflecting input from clinical and public health experts, to facilitate systematic, detailed, data collection.
